# Placental Related Disorders of Pregnancy

**DOI:** 10.3390/ijms23073519

**Published:** 2022-03-24

**Authors:** Eun D. Lee, Hiten D. Mistry

**Affiliations:** 1 Department of Microbiology and Immunology, School of Medicine, Massey Cancer Center, Virginia Commonwealth University, Richmond, VA 23298, USA; eun.lee@vcuhealth.org; 2 Department of Women and Children’s Health, School of Life Course Sciences, King’s College London, London SE5 9NU, UK

We are pleased to present this Special Issue of *International Journal of Molecular Sciences*, entitled ‘Placental Related Disorders of Pregnancy’. The placenta is a unique organ, produced outside the embryo and connected by a cord of vessels, and is formed as a result of various degrees of interactions between fetal and maternal tissues within the pregnant uterus. The placenta fulfils a variety of functions, which are completed by several different organs in adult life. Unlike the relatively stable mature adult organs, the placenta is programmed to complete very different functions during development. Thus, the placenta can be described as a constantly evolving organ. Its major role is the homeostasis of a protected environment for the undisturbed growth and development of an embryo/fetus.

Placental-related disorders of pregnancy are almost unique to the human species and affect around a third of human pregnancies. Many of these disorders result in increased maternal and fetal mortality and morbidity and can have life-long health implications for both the mother and her child. Recent changes in human lifestyle, such as delayed childbirth and hypercaloric diets, may have increased the global incidence of placental-related disorders over recent decades.

This Special Issue is a compilation of 21 research manuscripts and reviews, covering all aspects of placentation, with a particular focus on those related to placental function and disorders of pregnancy. The manuscripts cover aspects of placental physiology, biochemistry and molecular biology, and clinical and animal models are also included in this excellent Special Issue.

This collection contains some excellent reviews. The first review covers the homeostasis of the cytokine interleukin-15 (IL-15) in healthy pregnancy, providing up-to-date mechanisms of the action of IL-15 at the maternal–fetal interface [1]. A fascinating review by Anthony Carter covers why human placentation is so unique, with in-depth details on placentation in different animals to wonderfully illustrate this [2]. This is followed by a comprehensive review covering the important condition of gestational diabetes and the contribution of the placenta in the associated immunoendocrine dysregulation [3]. Finally, a very topical and informative overview highlighting the role of the placenta and the use of low-dose aspirin in the prevention of pre-eclampsia [4,5]. In addition to the reviews, our collection also contains several novel studies covering pre-eclampsia [6,7,8]; fetal growth restriction [7,9,10,11]; calcium signaling [12]; placental oxidative stress, nutrition, senescence and apoptosis [6,9,13,14,15]; sexual dimorphism [16,17,18], intrahepatic cholestasis [19]; placental vascular modelling [20]; and placental villous explant culture models [21].

This Special Issue presents placental research using a range of established and state-of-the-art techniques showcasing novel and up-to-date data to enhance and facilitate our understanding of placentation as well as mechanisms that result in associated adverse pregnancy outcomes, as well as longer-term risks of complications.
